# Activity Budget and Feeding Ecology of Geladas (*Theropithecus gelada obscurus*) around Abogedam Church West of Debre Berhan Town, Ethiopia

**DOI:** 10.1155/2020/9829834

**Published:** 2020-09-01

**Authors:** Dereje Yazezew, Afework Bekele, Hussein Ibrahim

**Affiliations:** ^1^Department of Biology, College of Natural and Computational Sciences, Debre Berhan University, P.O. Box 445, Debre Berhan, Ethiopia; ^2^Department of Zoological Sciences, College of Natural and Computational Sciences, Addis Ababa University, P.O. Box 1176, Addis Ababa, Ethiopia; ^3^Department of Biology, College of Natural and Computational Sciences, Wollo University, P.O. Box 1145, Dessie, Ethiopia

## Abstract

Geladas are the most distinctive of Ethiopian endemic mammals, representing the last extant species of primate genus that have a very restricted distribution in the northern Ethiopian plateau. The activity budget and feeding ecology of geladas (*Theropithecus gelada obscurus*) were studied around Abogedam Church, Ethiopia, from May to October 2014, encompassing dry and wet seasons. The scan sampling method was applied to collect behavioural data on the identified band. Activity scans were collected at 15-minute intervals for up to five minutes duration from 0700 to 1730 h. The activity recorded for each individual was the first activity that lasts for five seconds. During each scan, individuals were recorded as performing activities: feeding, moving, resting, playing, aggression, grooming, sexual activity, and others. On average, geladas devoted 57.19% feeding, 14.82% resting, 14.92% moving, 4.83% playing, 2.53% aggression, 4.14% grooming, 1.23% sexual activity, and 0.34% other activities such as vocalization, defecation, and urination. Forty-one plant species were consumed by geladas that belonged to 18 families of which 53.66% were grasses. This study provides basic information on further studies and motivates conservationists to plan the management of unprotected areas at the vicinity of agricultural lands where such endemic animals dwell.

## 1. Introduction

Ethiopia is blessed with diverse wildlife due to its geographical location and physical features [1, 2]. This is mainly reflected by the altitudinal range and the diversity of climate and vegetation, which are sources of endemism in both fauna and flora. Many of these endemic species are specifically associated with the high-altitude moorland and grassland habitats. Others belong to the highland forests while a few occur in the lowland forests of southwest Ethiopia [3]. Currently, there are about 315 species of mammals in Ethiopia, of which about 36 (11.4%) are endemic [4].

With increasing natural and anthropogenic impacts on wildlife, Ethiopia has many endemic primate species/subspecies where very few countries can claim. The Bale monkey (*Chlorocebus djamdjamensis*), Boutourlini's blue monkey (*Cercopithecus mitis boutourlinii*), colobus monkey (*Colobus guereza guereza* and *C. g. gallarum*), and gelada *(Theropithecus gelada)* are primate species/subspecies unique to the country. Particularly, the country is fortunate to have an endemic Cercopithecine genus and species, the gelada (*T. gelada*) [5], which is the subject of the present study.

Historically, nonhuman primates shifted their foraging ecology and survived with a new adaptation in a modified habitat. It has been believed that a series of environmental changes that transformed tropical forests into savannah woodlands [6] pushed frugivore primates to face longer periods of reduced food abundance. This forced them to use alternative sources such as meat, nuts, cereals, or underground storage organs of plants [7]. Forest nonhuman primates are thought to be particularly vulnerable to local extinction in fragmented landscapes [8]. Some arboreal primates were often unable to cross nonforest areas, which resulted in low population densities and often subjected to direct human persecution [9]. Consequently, species that are unable to adapt to modified habitats were forced to occupy small, marginal habitat patches. In small and fragmented populations, genetic diversity may be reduced owing to increased levels of drift and inbreeding. This reduced diversity is often associated with decreased fitness and a higher threat of extinction [10]. As a result, the long-term survival of many of these animals is questionable. Tangible examples that end up in extinction failing to adapt to the habitat modifications are the particular habitat specialists like *Theropithecus oswaldi*, *T. brumpti* and *T. darti* [11].

Geladas as one of the most distinctive of the Ethiopian endemic mammals [3], are Old World monkeys representing the last extant species of a primate genus [12]. This genus is an offshoot of the common African baboon lineage (genus *Papio*) that left the confine of its ancestral wooded habitats and inhabited the savannah during the late Miocene and early Pliocene [12, 13]. However, the genus had become extinct leaving the gelada on the remote high-altitude grassland of Ethiopia as its sole surviving representative.

*T. gelada* has a very restricted distribution, mainly in the northern part of the main (western) Ethiopian plateau. It occurs in the provinces of Tigray, Wollo, Gondar, and Shoa between latitudes 9° and 14° N in rocky gorges and precipices at altitudes between 2350 and 4400 m asl [14–18]. According to Gippoliti [19], the gelada distribution is still imperfectly known at the moment, and apparent gaps in its distribution range warrant further field research.

At present, there are two subspecies of geladas *T. gelada gelada* occurring north of Lake Tana, west of Tekezzie River and *T. g. obscurus* found southeast of Lake Tana, East of Tekezzie River [1, 20]. However, a third undescribed subspecies, *T. gelada senex,* is also located in the extreme south of the Rift valley, on the bank of Wabi Shebelle River [21–23].

Identifying how animals divide their activities throughout the day and year-round offers clear perception into their interaction with the environment and their strategies for maximizing energetic and reproductive success [24]. Activity budgets of primates are commonly associated with strategies of energy conservation [25, 26] and are affected by a predator or human pressure, social structure, season, or availability, distribution, and quality of food resources [27–34]. Primates may respond quite differently to forest disturbance, fragmentation, and related edge effects, which make the long-term survival of a particular primate species in an altered habitat depending on its specific habitat requirements [8, 35]. Hence, knowledge of the basic quantitative natural history of primate species is indispensable to their conservation [36]. For instance, data on feeding ecology provide information on food species and level of dietary specialization, while the data on activity budget assist in developing monitoring strategies for threatened and elusive primates by focusing on counting efforts when these primates are most active [37, 38].

Although the population and behavioural ecology of geladas in protected areas has been the subject of many foreign and Ethiopian researchers, the population and behaviour of geladas are not properly studied in unprotected human-dominated habitats due to inaccessibility of the areas [19]. As a result, this study aims to assess the activity budget and feeding ecology of geladas outside protected areas to determine whether these variables are similar or different from geladas in protected areas. In so doing, the study helps to understand the nature of the environmental factors such as the disturbance levels, foraging opportunities, food availability, and habitat quality of the species for future management and conservation efforts, and the sustainability of the study area biodiversity. We based our assumption on the following two hypotheses: (1) Geladas budget equal time for feeding during the dry and wet seasons. (2) Both underground and aboveground food resources are equally important for geladas.

## 2. Materials and Methods

### 2.1. Study Area

**Figure 1:**
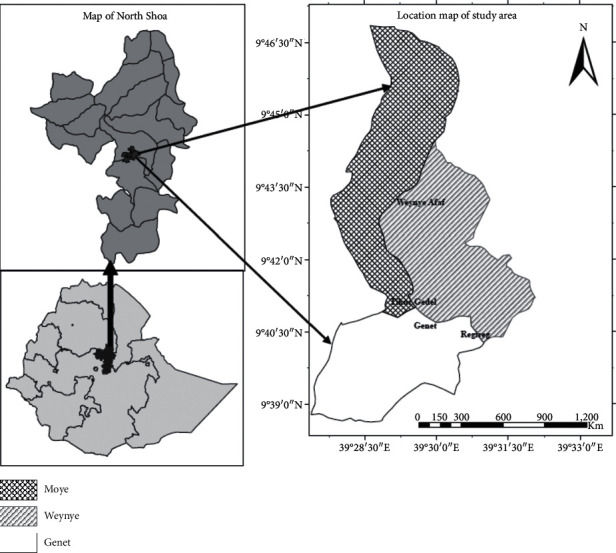
Map of the study area.

The present study was carried out around Abogedam Church near Debre Berhan Town, which is located in the North Shoa Zonal Administration of Amhara Regional State, northwestern highlands of Ethiopia. The area is situated approximately between 9°40′-9°44′N latitude and 39°28′-39°32′E longitude about 135 km north of Addis Ababa and lies west of the main road leading from Addis Ababa through Debre Berhan to Dessie (Figure 1). The altitude ranges between 2735 and 2847 m asl. The topography of the area is steep and dissected by ravines and gorges through which rivers and streams flow, eventually joining the Blue Nile Gorge. The numerous narrow shallow river valleys originate in the mountain ranges of the area, merging to form broader valleys, approximately 1 km wide and join deep steep sided river gorges before joining the Blue Nile.

**Figure 2:**
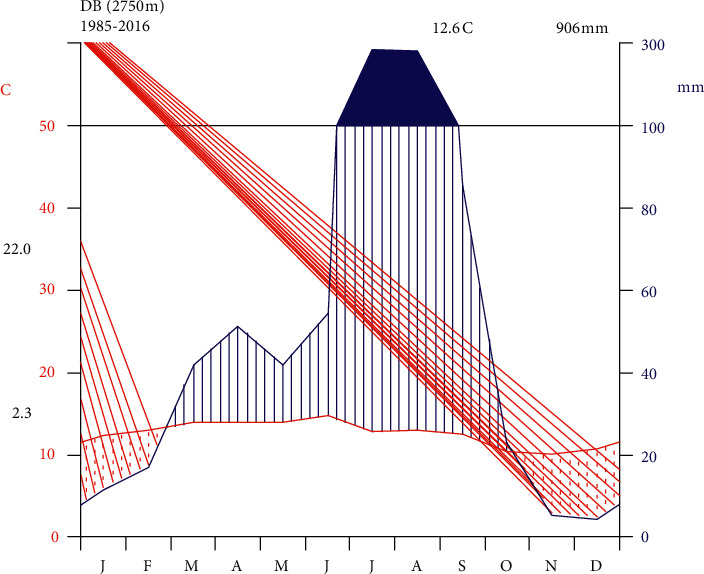
Climate diagram for the study area-town of Debre Berhan (DB), 2750 m asl (source: ©NMA, 2016).

The annual pattern of rainfall in the area is bimodal with a long rainy season during July-September (big rain-summer) and a short rainy season from February to May (small rain-spring) with the peak in April. Based on 32 years of climate data (1985–2016) obtained from the National Meteorological Agency (NMA), the mean annual temperature ranges between 2.3°C and 22°C, whereas the mean annual precipitation is 906 mm [39] (Figure 2). Since the area lies between elevations of 2735 and 2847 m asl, it has alpine climatic conditions. Geologically, the area lies on the Tertiary volcanic of the Ashangi group. Thin residual soils overlie these rocks, becoming thicker and coarser grained in the valleys. The Ashangi group is composed of alkaline basalts interbedded with pyroclastics and rhyolites [40]. The River Berresa flows down to join the Blue Nile throughout the year.

The total human populations of the area that directly or indirectly interact with gelada are 4087 people. Like any other part of the highlands of the country, mixed cultivation of livestock rearing and crop production are the main economic activities of the community. As this study on wildlife ecology is the pioneer in the study area, data are lacking that show the population trend of geladas and the diversity of other mammal species in the area. In addition to the endemic geladas, there are also a variety of wildlife populations in the study area including grivet monkey, common duiker, crested porcupine, Abyssinian hare, rock hyrax, spotted hyaena, common jackal, serval, honey badger, and different bird species. The vegetation type of the study area is mainly scattered shrubs interspersed with annual and perennial herbs in the sloppy area. The main plant species in the area are *Maytenus arbutifolia* (A. Rich.) Wilczek*, Juniperus procera* Hochst ex Endl., *Aloe* spp., *Dodonaea angustifoli*, *Opuntia ficus-indica* (L.) Miller, *Dovyalis abyssinica* (A. Rich.) Warb*., Carissa edulis* L., *Rosa abyssinica* Lindley, and *Oliniar ochetiana (*A. Juss.).

The local people in the study area are engaged in intensive agriculture, extending up to the edge of the cliff. Moreover, the communities alternate the landscape by modifying the shrubland, thereby changing the travel corridor to farmland which restricts pathways between groups in a population of geladas. Similar to other anthropogenic pressures, livestock grazing has strong impacts on geladas feeding ground and overall ecosystem functioning that forced the animals to obtain their forage resources from the remaining poor quality forage [41].

### 2.2. Preliminary Survey

A reconnaissance survey and habituation of a band of geladas to human observer were conducted on foot in the study area in April 2014. As geladas are familiar with humans, their behavioural activity is not disturbed with human presence and needs a shorter time to habituate them, especially for band identification. Some individual members that have unique natural markings such as swelling body parts and other identifiable features were used to identify the study band. The swell is permanent and its cause is obscure which needs further enquiry. Actual data collection was carried out from May to October to represent the wet and dry seasons.

### 2.3. Activity Time Budget

Behavioural data on the activity time budget for the identified band was collected using a scan sampling method for five consecutive study days per month [42–45]. During the scan sampling, activities of geladas were collected at 15-minutes intervals for up to five minutes duration from 0700 to 1730 h. The activity recorded for each individual was the first activity that lasts for five seconds [46, 47]. Intense attention was given to pinpoint scanned individuals in such a way that the researchers avoid scanning the same gelada more than once in a given scan [47]. During each scan, activity was recorded by scanning the group from left to right or vice versa to avoid possible bias towards eye-catching activities involving grooming, fighting, mating [43, 45, 47], and gum protrusion. The identity of the scanned individual was recorded and assigned to one of the following age/sex classes: adult male, adult female, subadults, and young. However, infants were excluded from scan sampling [43–48]. The following behavioural categories were recorded: feeding, moving, resting, playing, aggression, grooming, sexual activity, and others (urination, defecation, vocalization, and drinking) [43, 45, 47].

Feeding was recorded when individuals were foraging, grazing, digging, and transferring to the mouth or chewing food items. Moving was recorded when geladas were in quadrupedal locomotion such as walking, jumping, running, or climbing that resulted in changing of spatial position and not engaged in feeding or any form of social activity [43, 49]. Resting was recorded when the animal was inactive and either lying down, sitting, immobile in a quadrupedal stance, or self-grooming. Playing included hitting, biting, chasing, and other vigorous activities accompanied by movements and gestures by more than one individual interacting in a nonaggressive behaviour [43]. Aggression was recorded when a gelada was chased, bit, grabbed, displaced, and threatened by another gelada. Grooming was recorded when one gelada used its hands to discover or to clean the body of another gelada. Sexual activity was recorded when a gelada groomed the sexual organs, presenting, embracing, copulating, or engaged in mating activity. Other activities included vocalization, defecation, urination, drinking, and activities that do not fit in any of the other categories.

The percentage of engagement in different activities was calculated by dividing the proportion of the number of behavioural records for each activity category by the total number of activity records. The behavioural records of the band were then used to calculate the activity budgets per day and averaged within each month to construct monthly and seasonal activity budgets.

### 2.4. Feeding Ecology

During activity scan sampling, when geladas were observed feeding, the species as well as the type of food items consumed (long grass blades, short grass blades, herb leaves, herb roots, grass roots, corms, unidentified tubers, crops, animal prey, or others) were recorded. As geladas were observed feeding, the type of food item was recorded on a standardized data sheet [44, 50–53]. Known and identified food species consumed by members of geladas were recorded in the field and unidentified species were collected, named by their local name, pressed, and taken to Addis Ababa University National Herbarium for further taxonomic identification. The daily food items and species consumed by the group were summed up within each month to construct a monthly proportion of food items and food species consumed to compare seasonal food composition. The dietary composition was evaluated by determining the proportion of different dietary items and plant species based on the total number of records on the feeding band [45, 54].

### 2.5. Data Analysis

Statistical Package for Social Science (SPSS) 20.0 software for Windows Evaluation version was used to analyze the data collected during the survey. The nonparametric Pearson chi-square test was used to analyze the difference in the number of records for different activities at different seasons. For descriptive analysis of feeding records, plant species and food items consumed by geladas were used to identify the feeding behaviour of the species. The difference in feeding records for different food items was also analyzed by using Mann–Whitney *U* test. All statistical tests were two-tailed with 95% confidence intervals and level of rejection set at *P* = 0.05.

## 3. Results

**Figure 3:**
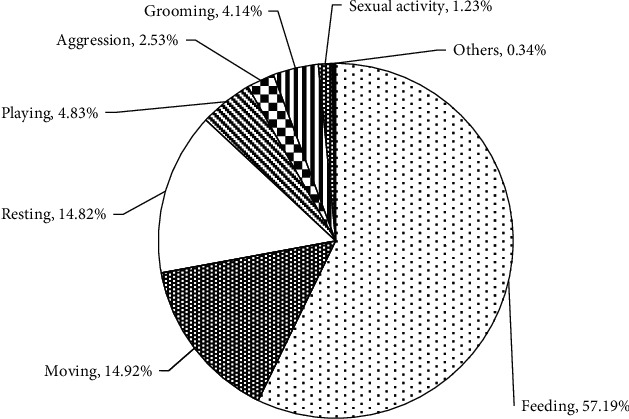
Overall activity time budget of geladas.

A total of 2922 individual behavioural observations were recorded from 840 group scans during 210 hrs. On average, geladas were observed 57.19% feeding, 14.82% resting, 14.92% moving, 4.83% playing, 2.53% aggression, 4.14% grooming, 1.23% sexual activity, and 0.34% in other activities (Figure 3).

**Figure 4:**
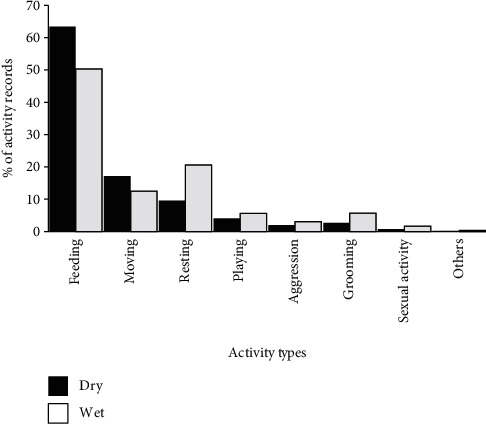
Seasonal activity budget of geladas.

Geladas, on average, engaged more in feeding (63.41%) during the dry season than the wet season (50.29%). There was a significant difference in feeding records between dry and wet seasons (*χ*^2^ = 45.92, *df* = 1, *P* < 0.05). Geladas were involved more in moving (17.12%) during the dry season than the wet season (12.48%). There was a significant difference in moving activity between dry and wet seasons (*χ*^2^ = 18.58, *df* = 1, *P* < 0.05). There was also a significant difference between seasons in resting (*χ*^2^ = 44.62, *df* = 1, *P* < 0.05), grooming (*χ*^2^ = 12.57, *df* = 1, *P* < 0.05), and sexual activities (*χ*^2^ = 4.0, *df* = 1, *P* < 0.05). However, there were no significant differences between seasons in playing (*χ*^2^ = 1.56, *df* = 1, *P* > 0.05), aggression (*χ*^2^ = 1.35, *df* = 1, *P* > 0.05), and other activities (*χ*^2^ = 0.4, *df* = 1, *P* > 0.05) (Figure 4).

A total of 1671 feeding behavioural observations were recorded from the scan sampling of geladas during the study period: 974 (dry season) and 697 (wet season). Grasses were the most consumed food items (65.1% grass blades and stems, and 6.45% grass roots) during the study periods (Table 1). Out of the plant species used by geladas, 65.1% were grass blades and stems, and 25.87% were tubers and roots (1.35% herb root, 6.45% grass roots, 15.46% tuber, and 2.61% unidentified tubers). There were significant differences in feeding records on grass blades and stems between dry and wet seasons (dry season 52.1% and wet season 77.91%; *P* < 0.05). However, there was no significant difference in feeding records on unidentified tubers (*P* > 0.05) and cereals (*P* > 0.05) between seasons. Geladas carried out feeding chiefly in a seated position and the hand actions used are primarily related to harvesting the grass blades, leaves, flowers, fruits, animal preys, and tubers.

During the present study, geladas consumed a total of 41 plant species belonging to 17 families. The highest contribution of the diet was from the family Poaceae comprising 53.66% of dietary plant species (Table 2).

## 4. Discussion

So far, research on gelada has been restricted mainly to the protected areas marginalizing those populations occupying unprotected areas. Accordingly, the results of this study are compared with studies carried out on protected areas of the country.

The activity time budget of geladas fluctuates on an hourly and daily basis in response to environmental variables, the most important of which are food availability and quality, which can also be affected by human disturbances [44]. In accordance with studies by Dunbar [55], Hunter [53], and Kifle et al. [44], geladas exhibited themselves to feeding over other activities in both seasons. This is probably due to the consequence of bulk feeding adaptation emanating from their highly specialized graminivorous diet [56]. However, this is contrary to observation with Abu et al. [57] on Arsi geladas where geladas exhibited themselves on feeding about 42%. Geladas forage more during the dry season than the wet season, which is in line with the reports of Hunter [53], Iwamoto [58], and Kifle et al. [44]. There were significant differences between seasons in activity records on feeding, resting, moving, sexual activity, and grooming but not in playing and aggression. The plausible reason for more moving activity during the dry season might be associated with scarce resources and the quality of food and availability of free-roaming farmland areas as farmers harvest their crops and leave the bare agricultural land. Usually, dry season affects forage availability where fleshy grasses and other food sources are scarce and leads geladas to travel more and search for food to satisfy their nutritional demands. Iwamoto and Dunbar [56] stated that feeding increases in response to the decrease in the protein content of the dry season forage, and hence feeding activity would increase with respect to the nutritional requirements.

During the wet season, however, green grasses were plenty and geladas satisfy their energy demand. As a result, they were devoted more to resting and sexual activities. Accordingly, geladas move less during the wet season. This finding is in line with the findings of Dunbar [55] in the Sankaber area of the Simien Mountains National Park and Kifle et al. [44] in Wonchit Valley, Ethiopia.

Although grasses accounted for more than 71% of gelada's diet throughout the course of this study, our results differ from the observations made by Dunbar [55] (90%), Hunter [53] (85.6% for wet season), Iwamoto and Dunbar [56] (92.1%), and Kifle et al. [44] (83.7%), where grass accounted for more than 83% of geladas diet. The plausible reason for the lower proportion of grasses in the diet of geladas in this study might be attributed to the relative food scarcity of the most favoured dietary items due to intensive habitat disturbances outside protected areas. Moreover, there was a considerable seasonal variation in the parts of grasses consumed. Geladas feed on grass blades and stems 52.1% and 77.91% during dry and wet seasons, respectively. Feeding intensity on other plants is also dependent on seasonal availability.

The data showed a marked difference in the proportion of feeding activity on grass blades and roots, tubers, and herb leaves in response to the seasonal availability of food items [44, 58]. When fresh green grass is abundant everywhere during the wet season, geladas consumed a small amount of other food items. When the availability of preferred grass blades is limited during the dry season and ample nutrition is available below the ground, geladas concentrated more heavily on tubers and roots [44, 55, 58]. Geladas feed on grass blades only as long as they remain green, preferring instead to dig for roots and rhizomes during the dry season once the grasses become desiccated and less digestible [14, 55, 56, 59]. As feeding activity increases in the low-quality habitat of an area to compensate for the energy demand [53], geladas feed more on these underground resources, which were not used by other ungulate competitors during the dry season. Geladas feed on tubers, leaves and roots of herbs, grass roots, and animal preys such as termites and ants more during the dry season than the wet season. Accordingly, these food items can be considered as fallback foods as geladas utilize them when preferred green grass blades (leaves) are unavailable [60, 61]. We attributed this due to that geladas are unique among the primates in their exploitation of the graminivorous (grass-eating) niche [59].

## 5. Conclusion

This study offers information on different aspects of the ecology of geladas outside protected areas. The study provides baseline information for further studies on this species in the current study area and other protected and unprotected areas. Moreover, the study ignites conservation demands on stakeholders to correlate directly to the management of unprotected areas at the vicinity of agricultural lands where such endemic wildlife dwell. Wide ranges of factors such as climatic conditions, food availability and quality, and human disturbances influence the diurnal activity budget of geladas in the study area. Geladas are devoted more to feeding activity than other activity types in both dry and wet seasons to compensate for the high energy demand. Geladas consume various plant species ranging from grass to shrub species with varying amounts on a seasonal basis. Moreover, they feed on different parts of the plant species, with different degrees of preferences, although they feed on less preferred food when there is scarcity. The following points are recommended to enhance the value of this unprotected area for the future survival of geladas:

- Stakeholders in the area and government officials should work towards designing a fertile ground for ecotourism as the area is nearer to Debre Birhan town.
- Monitoring of this charismatic species should be taken as a priority component of wildlife management plans. So that geladas attract tourists to the area and become a national treasure, which might outweigh the economic value of crops and livestock.
- Subsequent studies should be conducted to determine the trajectory of a population and behavioural ecology of geladas in the area.

## Figures and Tables

**Table 1:** Percentage composition of food items consumed by geladas during the wet and dry seasons.

Plant part	Dry	Wet	Mean
Grass blade and stems	52.1	77.91	65.01
Herb leaves	3.28	6.74	5.01
Herb roots	2.26	0.43	1.35
Grass roots	11.6	1.29	6.45
Tuber	23.31	7.6	15.45
Unidentified tuber	2.76	2.44	2.61
Cereals	0.4	0.29	0.35
Animal preys	3.68		1.85
Others	0.61	3.3	1.96

**Table 2:** List of plant species, parts consumed, and percentage composition of diet consumed by geladas during the study period.

Family name	Scientific name	Habit	Food items consumed	Seasons	
				Wet	Dry
Aloaceae	*Aloe* spp.	Herb	FL		x
Anacaridaceae	*Rhus vulgaris* Meikle	Shrub	FR		x
Anthericaceae	*Chlorophytum tetraphyllum (*L.f.) Baker	Herb	TU		x
Anthericaceae	*Chlorophytum pterocarpum* Nordal & Thulin	Herb	TU		x
Apocynaceae	*Carissa edulis* Wahl.	Shrub	FR	x	
Asteraceae	*Haplocarpha schimperi* (sch. Bip.) Beauv.	Herb	RO	x	
Asteraceae	*Crepisru eppellii* sch. Bip.	Herb	Le	x	
Cactacea	*Opuntiaficus-indica* (L.) Miller	Shrub	LE, FR		x
Colchicaceae	*Merendera abyssinica* A. Rich.	Herb	TU		x
Cyperaceae	*Cyperus rigidifolius* Steud.	Herb	TU		x
Fabaceae	*Vicia faba* L.	Herb	LE	x	
Fabaceae	*Trifolium temnense* Fresen	Herb	LE	x	
Flacourtiaceae	*Dovyalis abyssinica* (a. Rich.) Warb.	Shrub	FR		x
Iridaceae	*Romulea fischeri* Pax	Herb	TU		x
Lamiaceae	*Thymus schimperi* Ronniger	Herb	LE	x	
Onagraceae	*Epilobium hirsutum* L.	Herb	LE, FL	x	
Plantagiaceae	*Plantago lanceolate* L.	Herb	TU	x	
Poaceae	*Cynodon aethiopicus* Clayton & Harlan	Grass	BL, RO	x	x
Poaceae	*Cynodon dactylon* (L.) Pers.	Grass	BL, RO	x	x
Poaceae	*Andropogone abyssinicus* Fresen	Grass	BL, RO	x	x
Poaceae	*Andropogone chinensis* (nees) Merr.	Grass	BL	x	x
Poaceae	*Andropogone gayanus* Kunth	Grass	BL	x	x
Poaceae	*Andropogone chrysostachyus* Steud	Grass	BL	x	x
Poaceae	*Andropogone fastigiatus* Sw.	Grass	BL	x	x
Poaceae	*Pennisetum thunbergii* Kunth	Grass	BL	x	x
Poaceae	*Pennisetum glabrum* Steud	Grass	BL	x	x
Poaceae	*Pennisetum sphacelatum* (nees) Th. Dur. & Schinz	Grass	BL	x	x
Poaceae	*Pennisetum clandestinum* Chiov	Grass	RO	x	x
Poaceae	*Pennisetum thunbergii* Kunth	Grass	BL	x	x
Poaceae	*Sporobolus pyramidalis* Beauv	Grass	BL	x	x
Poaceae	*Themeda trianda* Forssk	Grass	BL	x	x
Poaceae	*Hyparrhenia rufa* (nees) Stapf.	Grass	BL	x	x
Poaceae	*Eragrostis tenella*P. Beauv. ex Roem. & Schult.	Grass	BL	x	
Poaceae	*Eragrostis lepida* (a. Rich.) Hochst. ex Steud.	Grass	BL	x	
Poaceae	*Digitaria ternata* (a. Rich.) Staf	Grass	BL	x	
Poaceae	*Digitaria abyssinica* (Hochst.ex A. Rich.) Stapf	Grass	BL	x	
Poaceae	*Eleusine floccifolia* (forssk.) Spreng.	Grass	BL	x	x
Poaceae	*Hordeum vulgare* L.	Herb	FR	x	
Poaceae	*Triticum* spp.	Herb	FR	x	
Rosaceae	*Rosa abyssinica* Lindley	Shrub	FR	x	x
Solanaceae	*Physalis peruviana* L.	Herb	FR	x	

BL = grass blade, TU = tuber, RO = root, LE = leaves, FR = fruits, and FL = flower.

## Data Availability

The data used are available from the corresponding author upon request.
